# Effect of temperature on the biological parameters of pink bollworm, *Pectinophora gossypiella* Saunders (Lepidoptera: Gelechiidae)

**DOI:** 10.1038/s41598-024-65241-8

**Published:** 2024-07-01

**Authors:** Madhu Tadagavadi Nagaraju, Kamanur Murali Mohan, Manikyanahalli Chandrashekara Keerthi, Tenguri Prabhulinga, Shivaji Thube, Vivek Shah, Hosam O. Elansary, Ihab Mohamed Mousa, Mohamed A. El-Sheikh

**Affiliations:** 1 ICAR-Central Institute for Cotton Research, Nagpur, 440010 Maharashtra India; 2 ICAR-Central Plantation Crops Research Institute, Regional Station, Vittal, 574243 Karnataka India; 3 University of Agricultural Sciences, Bangalore, 560065 Karnataka India; 4 ICAR-Indian Institute of Horticultural Research, Bengaluru, 560089 Karnataka India; 5 Department of Plant Production, College of Food and Agricultural Sciences, King Saud University, P.O Box 2460, 11451 Riyadh, Saudi Arabia; 6 Department of Botany and Microbiology, College of Food and Agricultural Sciences, King Saud University, P.O Box 2455, 11451 Riyadh, Saudi Arabia; 7 Department of Botany and Microbiology, College of Sciences, King Saud University, P.O Box 2455, 11451 Riyadh, Saudi Arabia

**Keywords:** Developmental biology, Ecology, Zoology, Climate sciences, Environmental sciences

## Abstract

Pink bollworm (PBW) *Pectinophora gossypiella* is an important pest cotton worldwide. There are multiple factors which determines the occurrence and distribution of *P. gossypiella* across different cotton growing regions of the world, and one such key factor is ‘temperature’. The aim was to analyze the life history traits of PBW across varying temperature conditions. We systematically explored the biological and demographic parameters of *P. gossypiella* at five distinct temperatures; 20, 25, 30, 35 and 40 ± 1 °C maintaining a photoperiod of LD 16:8 h. The results revealed that the total developmental period of PBW shortens with rising temperatures, and the highest larval survival rates were observed between 30 °C and 35 °C, reaching 86.66% and 80.67%, respectively. Moreover, significant impacts were observed as the pupal weight, percent mating success, and fecundity exhibited higher values at 30 °C and 35 °C. Conversely, percent egg hatching, larval survival, and adult emergence were notably lower at 20 °C and 40 °C, respectively. Adult longevity decreased with rising temperatures, with females outliving males across all treatments. Notably, thermal stress had a persistent effect on the F1 generation, significantly affecting immature stages (egg and larvae), while its impact on reproductive potential was minimal. These findings offer valuable insights for predicting the population dynamics of *P. gossypiella* at the field level and developing climate-resilient management strategies in cotton.

## Introduction

Pink bollworm (PBW) *Pectinophora gossypiella* Saunders (Lepidoptera: Gelechiidae) is an economically important pest of cotton and native to the Indian subcontinent^1^. The spread of this pest throughout the cotton-growing regions of world has been documented for the last two centuries^2^. In India, the *P. gossypiella* attained the status of destructive pest and can be found in all cotton growing areas, causing up to 68 percent yield loss and 37.5 percent locule damage in non-*Bt* and *Bt* cotton cultivars, respectively^3,4^. It is a stenophagous pest, has a restricted host range, but primarily adapted to the genus *Gossypium* spp; however, it also feeds and oviposits occasionally on other species of Malvaceae, but not known to build pestiferous populations^5,6^. Being an internal feeder, the larvae spent most its time inside the boll by feeding on seeds; hence, it is difficult to control through conventional approaches^7,8^. Due to this the widespread infestation of *P. gossypiella* was recorded across cotton growing regions of India due to multiple factors including resistance to *Bt* toxins, less effectiveness of insecticides, changing climatic conditions and others^9–11^.

The effect of climate change on the agriculture sector is a major concern. Global temperature is expected to increase by 1.5–4.5 °C by the end of the century^12^. The physiological response of species to abiotic factors such as temperature, rainfall, humidity and photo-period has gained considerable attention in the scientific community for more than a century. Of these, temperature is one of the important variables of the environment that can influence phenotypic plasticity and affect various aspects of the life history parameters of insects^13^. The distribution and abundance of pests in an ecosystem are largely influenced by temperature^14^. Insects may undergo bio-physical changes like the accumulation of sugars and varying the fluidity of cellular membranes in accordance with temperature^15^. The increased metabolic rate of an organisms corresponding with increased temperature subsequently results in a higher growth rate with a short developmental time^16^. However, the rate of changes in development of insects occurs at specific temperature range; even a small variation makes significant changes in the duration of the life cycle and ultimately reflects in its fitness^17^.

The life history parameters of insects, including developmental time, fecundity and longevity are of great importance for their survival^18^. Temperature greatly influences the duration of each instar of larvae, resulting in physiological changes in the development of gonads before reaching the adult stage. In addition, faster development can be more advantageous for an insect to escape from predation or parasitism^19^. In recent years, the severe infestation of *P. gossypiella* was observed in the central and northern parts of India; the outbreak of this pest was affected by changes in climatic factors. However, the information regarding thermal effects on the life history traits of *P. gossypiella* and its transgenerational effect is minimal. In this context, it is crucial to investigate the effect of temperature on the life parameters of *P. gossypiella* under the current changing climatic conditions. In the present study, we systematically investigated the developmental and reproductive traits under six different temperatures to evaluate the thermal fitness of *P. gossypiella.*

## Results

### Developmental time and survival rate

**Figure 1:**
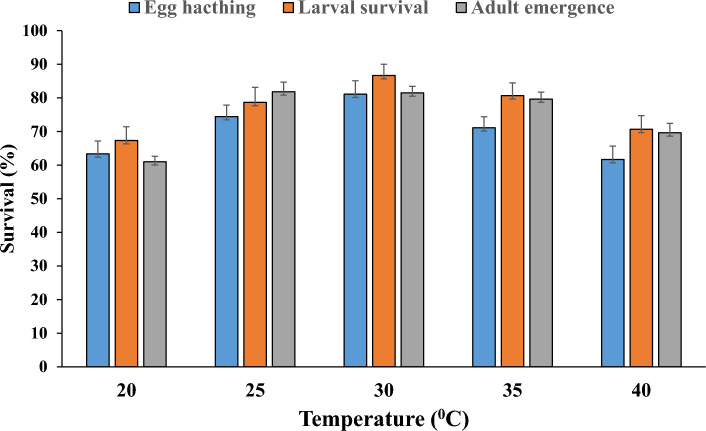
The percent egg hatchability, larval survival and adult emergence of P. gossypiella at five different temperature.

The developmental stages of *P. gossypiella* from egg to pupa were significantly influenced under different thermal regimes (Table 1). The incubation period decreased substantially when exposed to a temperature of 20 to 40 ^o^C (*F* = 44.83, *p* < 0.001). Similarly, the larval developmental time decreased as temperature increased at each instar (*F* = 29.51, 66.81, 60.37, 69.38, *p* < 0.001), and the total duration of larvae also differed significantly at respective temperatures. The developmental time of egg, larvae and pupae were significantly longer at 20 ^o^C and shorter at 40 ^o^C; whereas, no significant difference was observed between 30 and 35 ^o^C (Table 1 and 3). Egg hatching and larval survival rate was lower at 20 and 40 ^o^C; but, they did not differ significantly at 25, 30 and 35 ^o^C (Fig. 1). The similar trend was also shown in the age-stage-specific survival rate (S_xj_) and age-stage life expectancy (e_xj_). Further, the duration of development of pupae varies from 7 to 11 days at different temperatures. Interestingly 13.33% (20 individuals) did not pupate at 20 ^o^C and underwent diapause at fourth instar; the total diapause period was ranged from 43 to 105 days, respectively.

**Table 1:** Mean duration of (± SE) each developmental stage of *Pectinophora gossypiella* under different temperatures.

Temperature (°C)	Developmental time (days)					
	Egg	I instar	II instar	III instar	IV instar	Pupa
20	4.97 ± 0.13a	5.13 ± 0.14a	6.60 ± 0.18a	7.01 ± 0.57a	7.91 ± 0.84a	11.12 ± 1.26a
25	4.86 ± 0.16ab	4.93 ± 0.13a	5.66 ± 0.17a	6.20 ± 0.38a	6.93 ± 0.63a	10.04 ± 1.28a
30	3.86 ± 0.12b	4.3 ± 0.15b	4.80 ± 0.16b	5.30 ± 0.25b	5.67 ± 0.43b	8.74 ± 1.17b
35	3.50 ± 0.57c	3.77 ± 0.11c	4.00 ± 0.12c	4.71 ± 0.21c	4.86 ± 0.42c	7.77 ± 1.13c
40	3.17 ± 0.70c	3.56 ± 0.50c	3.60 ± 0.10d	4.17 ± 0.42d	4.33 ± 0.41d	7.26 ± 1.11d
*p* value	0.0001	0.0001	0.0001	0.0001	0.0001	0.0001

Means (± SE) within one row followed by different letters are significantly different at the 0.01 level based on one-way ANOVA and Tukey’s HSD multiple tests.

### Pupal weight and adult emergence

**Figure 2:**
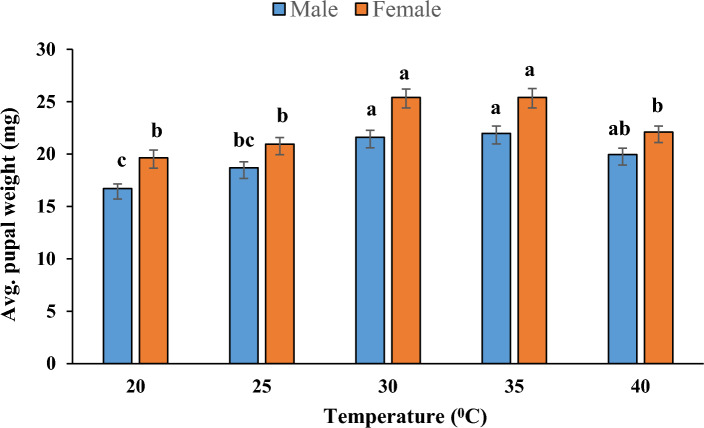
Male and female pupal weight of P. gossypiella at five different temperature.

The pupal weight of both male (*F* = 13.06, *p* < 0.001) and female (*F* = 16.93,* p* < 0.001) varied significantly at different temperatures and it was highest at 30 ^o^C (21.62 ± 0.66 and 25.42 ± 0.80 mg) and 35  ^o^C (21.97 ± 0.70 and 25.40 ± 0.85 mg), respectively and the lowest pupal weight was recorded at 20 ^o^C (16.70 ± 0.45 and 19.64 ± 0.72 mg) (Fig. 2). However, the pupal weight of females was comparatively higher than that of males in all treatments (*p* < 0.001). The regression coefficient of male and female pupal weight (*r* = 0.32, 0.50) showed a negative relation with temperature. Further, the percent adult emergence of *P. gossypiella* was also significantly affected at different temperature; the maximum adults emerged between 25 to 35 ^o^C and lower emergence was recorded at 20 and 40 ^o^C (Fig. 1).

### Reproductive parameters

#### Pre-oviposition and oviposition period

Temperature affected significantly on pre-oviposition and oviposition period of *P. gossypiella* (*F* = 10.06, 9.49, *p* < 0.001). The pre-oviposition and oviposition period decreased as temperature increased and it ranged from 2.12 ± 0.12 to 3.04 ± 0.15 and 5.56 ± 1.14 to 7.24 ± 1.26 days, respectively (Table 2).

**Table 2:** Adult longevity and reproductive parameters of *Pectinophora gossypiella* under different temperatures.

Temperature (°C)	Pre -ovipositional period (d)	Ovipositional period (d)	Mating success (%)	Fecundity/female	Sex ratio (F:M)	Adult longevity (days)	
						Male	Female
20	3.04 ± 0.15a	7.24 ± 1.26a	48 ± 6.16d	52.48 ± 2.88c	1.12 ± 0.02a	13.08 ± 1.49a	14.72 ± 1.33a
25	2.80 ± 0.14ab	6.56 ± 1.31ab	60 ± 5.48c	57.44 ± 3.17c	1.11 ± 0.02a	12.56 ± 1.34a	13.25 ± 1.48ab
30	2.36 ± 0.10bc	6.04 ± 1.17bc	84 ± 6.63a	77.92 ± 3.35ab	1.14 ± 0.03a	10.84 ± 1.28a	12.92 ± 1.44ab
35	2.32 ± 0.11c	5.68 ± 1.18c	80 ± 5.16ab	80.68 ± 2.81a	1.11 ± 0.02a	10.12 ± 1.31b	12.12 ± 1.04bc
40	2.12 ± 0.12c	5.56 ± 1.14c	72 ± 6.17b	70.12 ± 1.87b	1.10 ± 0.02a	9.92 ± 0.62b	11.36 ± 1.37c
*p* value	0.0001	0.0001	0.0001	0.0001	0.804	0.0001	0.0001

Values (± SE) within one row followed by different letters are significantly different at the 0.01 level based on one-way ANOVA and Tukey’s HSD multiple tests.

#### Mating success, fecundity, sex ratio and adult longevity

**Figure 3:**
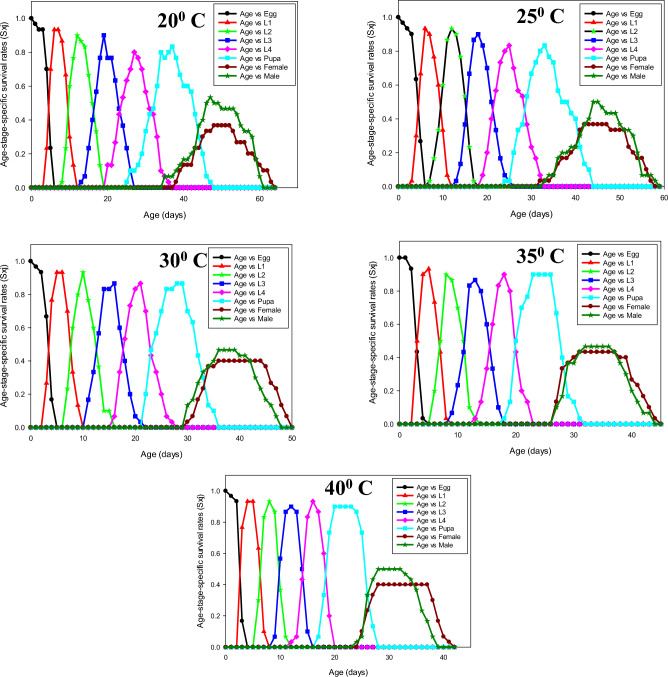
Age-stage-specific survival rate (Sxj) of each developmental stage of P. gossypiella at different temperature.

**Figure 4:**
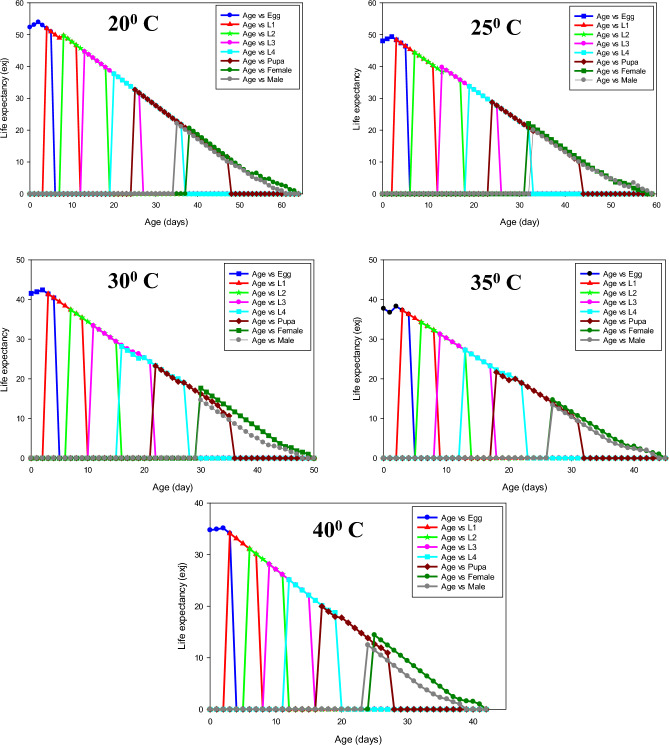
Age-stage life expectancy (exj) of each developmental stage of P. gossypiella at five different temperature.

**Figure 5:**
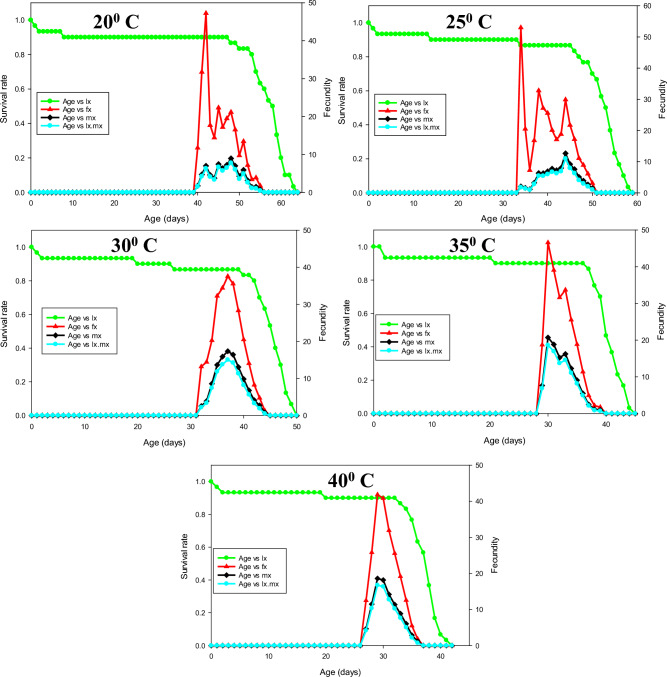
The age-specific survival rate (lx), female age-specific fecundity (fx), age-specific fecundity (mx), and age-specific maternity (lx.mx) of P. gossypiella at different temperature.

**Figure 6:**
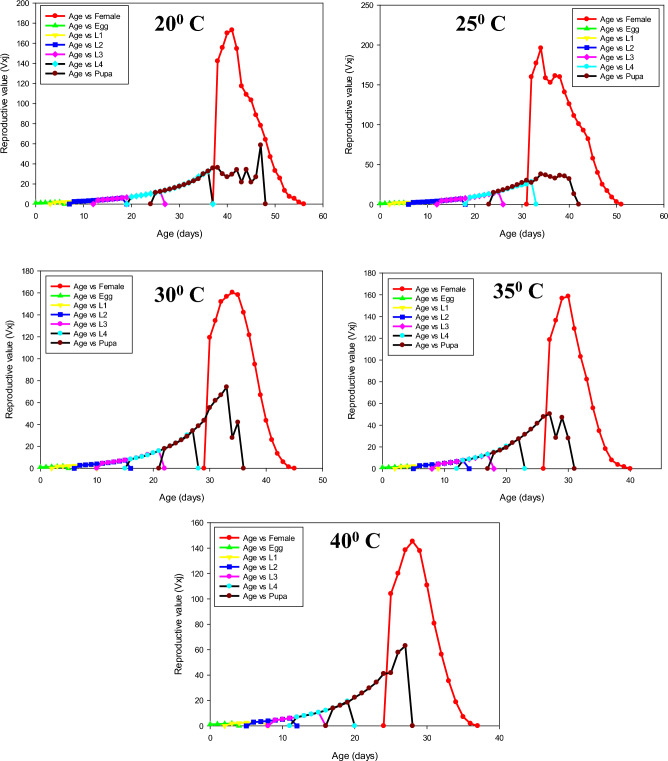
Age-stage reproductive value (Vxj) of P. gossypiella at five different temperature.

**Figure 7:**
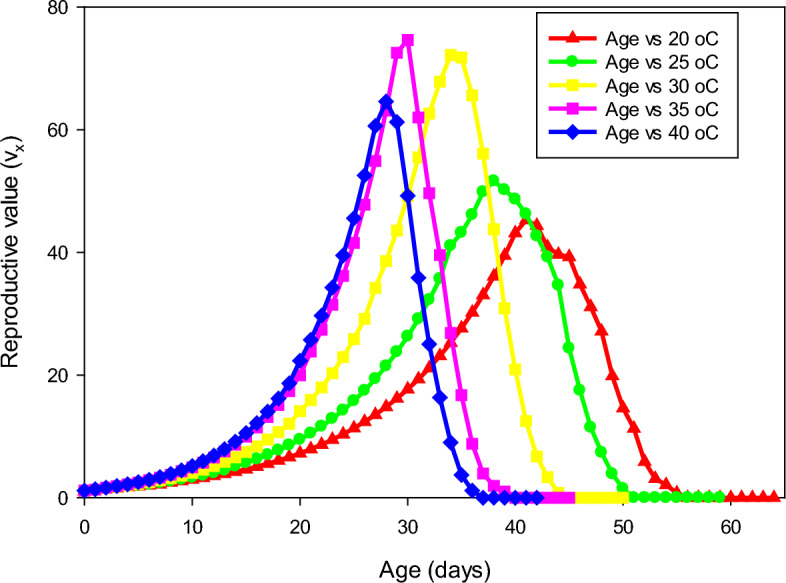
Reproductive value (Vx) of P. gossypiella at five different temperature.

The percentage of mating success of *P. gossypiella* showed significant differences and it was highest at 30 ^o^C (84 ± 6.63), followed by 35 ^o^C (80 ± 5.16). The individuals were exposed to 20 ^o^C exhibited less mating success (48 ± 6.16). The fecundity of *P. gossypiella* also differed significantly at respective temperatures (*F* = 30.83, *p* < 0.001). The highest fecundity was recorded at 30 and 35° C with a mean of 77.92 ± 3.35 and 80.68 ± 2.81 eggs/female, respectively, and lowest fecundity at 20 ^o^C (52.48 ± 2.88) (Figs. 3–7 and Table 2). However, no significant differences were recorded in the sex ratio at all temperatures (*p* < 0.804). The longevity of male and female adults (*F* = 15.48 and 9.17, *p* < 0.001) was significant, and it was varied from 9.92 ± 0.62 to 13.08 ± 1.49 in males and 11.36 ± 1.37 to 14.72 ± 1.33 in female adults, respectively. As the temperature increased, the longevity of both male and female adults were decreased. However, the longevity of females was higher than that of male adults in all the treatments. Furthermore, the intrinsic rates of increase (*r*), finite rates of increase (*λ*), net reproductive rates (R_0_), gross reproduction rate (GRR), mean generation times (T) and doubling time (DT) for various groups are shown in Table 3

.

**Table 3:** Population growth parameters of *P. gossypiella* at different temperatures.

Temperature (°C)	Intrinsic rate of increase (r)	Finite rate of increase (λ)	Net reproductive rate (R_0_)	Gross reproduction rate (GRR)	Mean generation time (T)	Doubling time (DT)
20	0.09 ± 0.001e	1.09 ± 0.001e	66.13 ± 1.573c	75.76 ± 1.737c	46.96 ± 0.111a	7.77 ± 0.063a
25	0.10 ± 0.001d	0.11 ± 0.001d	78.97 ± 1.752b	92.83 ± 1.963b	42.80 ± 0.106b	6.79 ± 0.046b
30	0.12 ± 0.001c	1.13 ± 0.001c	98.73 ± 1.970a	115.61 ± 2.242a	37.94 ± 0.054c	5.73 ± 0.029c
35	0.14 ± 0.001b	1.148 ± 0.001b	98.6 ± 2.153a	110.03 ± 2.163a	32.95 ± 0.040d	4.98 ± 0.026d
40	0.14 ± 0.001a	1.152 ± 0.001a	86.2 ± 1.690b	97.32 ± 1.803b	31.20 ± 0.032e	4.85 ± 0.024e
*p* value	0.0001	0.0001	0.0001	0.0001	0.0001	0.0001

Means followed by different letters in the same row are significantly different by using paired bootstrap test based on the CI of difference. Standard errors were estimated by using 100,000 bootstrap resampling.

#### Thermal effect on F_1_ generation

The persistence effect of thermal stress over the F_1_ generation of *P. gossypiella* on various biological parameters was recorded (Table 4). Similar to F_0_, the egg (F = 18.64, *p* > 0.001), and larval period (F = 10.65, *p* > 0.001) showed significant differences and the total duration decreased as temperature increased. However, no significant differences were recorded in egg hatching percentage and pupal weight, but it was slightly higher from the female parent exposed at 30 ^o^C (79.44 ± 3.03, 22.30 ± 0.78) than others. In addition, the percentage of successful mating and fecundity was comparatively higher from those reared at higher temperatures viz*.,* 30, 35 and 40 ^o^C. Further, significant differences were also recorded on the longevity of male (F = 4.05, *p* > 0.01) and females (F = 3.67, *p* > 0.01) adults. The longevity was comparatively higher at 20 ^o^C and female adults lived longer than males.

**Table 4:** Effect of thermal stress on life history traits of *P. gossypiella* over F1 generation.

Temperature (°C)	Incubation period (d)	Egg hatching (%)	Larval period (d)	Pupal period (d)	Pupal weight (mg)	Mating success (%)	Fecundity/female	Male longevity (d)	Female longevity (d)
20	4.44 ± 0.14a	66.67 ± 4.71a	23.44 ± 1.67a	10.16 ± 0.31a	20.34 ± 0.81a	68 ± 3.50a	63.60 ± 3.46c	12.04 ± 1.52a	13.92 ± 1.43a
25	4.32 ± 0.13a	75.56 ± 4.19a	20.28 ± 1.46b	9.56 ± 0.26ab	20.58 ± 1.69a	72 ± 4.16a	70.84 ± 3.60c	11.36 ± 1.49ab	12.52 ± 1.50ab
30	3.56 ± 0.12b	79.44 ± 3.03a	19.68 ± 1.35b	9.04 ± 0.27ab	22.30 ± 0.78a	88 ± 6.60a	73.46 ± 4.88ab	10.40 ± 1.38b	12.44 ± 1.44ab
35	3.44 ± 0.13b	68.89 ± 4.10a	20.12 ± 1.54b	8.92 ± 0.33b	21.56 ± 0.83a	84 ± 5.45a	73.52 ± 3.86a	10.16 ± 1.34b	12.04 ± 1.45b
40	3.28 ± 0.09b	63.34 ± 3.45a	19.24 ± 1.47b	8.96 ± 0.30b	20.14 ± 0.73a	88 ± 5.66a	74.16 ± 3.43b	10.12 ± 1.33b	11.68 ± 1.39b
*p* value	0.0001	0.048	0.0001	0.013	0.236	0.245	0.032	0.004	0.007

Values (± SE) within one row followed by different letters are significantly different at the 0.01 level based on one-way ANOVA and Tukey's HSD multiple tests.

## Discussion

*Pectinophora gossypiella* is a multivoltine, mostly stenophogous herbivore that feeds on cotton and other closely related species. The climate change has shifted the distribution of temperature variability and extremities which may impact the natural development of an organism and successful colonization^20^. In this context, understanding the life history traits of *P. gossypiella* is imperative to develop climate resilient management strategies. In the present study, *P. gossypiella* possesses the traits to become a successful colonizer species at varied temperatures. The mean duration of egg, larval and pupal development was substantially longer at 20 ^o^C and shorter at 40 ^o^C. However, the results from other studies were slightly inconsistent with our observation and reported that the duration of egg development was close to 10 days at 20 ^o^C and 4 days at 35 ^o^C, respectively^21,22^. Consequently, *P. gossypiella* took less time to complete a generation from egg to adult at 40°C with 26.09 ± 3.24 days compared to 42.74 ± 3.12 days at 20 °C, respectively. Hence, *P. gossypiella* can complete more generations in a season/year under high thermal regimes. However, this is mainly associated with metabolic reactions; many researchers reported that, an increase in temperature could hasten the process of metabolism at each level and produce more generations; therefore, the temperature is one of the important lifespan determinants in arthropods^23–26^. Furthermore, the percent egg hatchability and larval survival reached up to 81.2 and 86.7% at 30 °C; whereas, it was comparatively lower at 20 and 40 °C, indicating that any variation (low or high) from the normal range affects the developmental process; these results are consistent with some studies and they reported that, larval mortality of *Spodoptera frugiperda* (J.E. Smith) and *P. gossypiella* to be higher at 18 and 37 °C^27,28^. Similarly, based on the simulation model the temperature threshold and thermal requirement for *P. gossypiella* under field conditions was varied between 25 to 30 °C^22^.

In most cases, pupal weight determines the success of adult emergence, fecundity and adult longevity in holometabolous insects. In our study, the pupal weight was higher at 30 and 35 ^o^C; and a corresponding increase in adult emergence, mating success and fecundity was recorded. Our study supports the ‘fecundity advantage hypothesis’ (*i.e.,* the larger female produces more progenies) by producing more eggs from the females which were emerged from high pupal weight. Some studies reported that, the fecundity and fertility of *P. gossypiella* were significantly increased from those moths emerged from larger pupae; hence, pupal weight is a critical biological indicator of reproductive fitness in insects^29,30^. However, we found that the length of the pre-ovipositional and ovipositional periods decreases with increasing temperature. In addition, *P. gossypiella* exhibited excellent mating success up to 84 and 80% at 30 and 35 °C respectively; these results speculate that, adults may engage in copulation at the optimum temperature threshold and contribute more progenies for the next generation. Further, adult’s longevity decreased as temperature increased in both male and females with a difference of 3.16 and 3.36 days from 20 to 40 °C, respectively; however, females lived longer than males in all treatments. This may be associated with ‘heat flow’; where, a high rate of heat flow increases the rate of metabolism and reduces the lifespan; hence, metabolic rate is a crucial factor in determining longevity^31,32^. Further, the climatic variables such as precipitation, carbon-di-oxide, relative humidity together with temperature interact with plants in numerous ways and affects population dynamics of insect pests; as a result, expansion of host suitability and occurrence of new insect or invasive pests on various crops have been recorded over past century^33^.

The transgenerational effect of thermal stress on the biological parameters has been studied in some important lepidopteran insects like *S. litura*, *S. frugiperda*, *Helicoverpa armigera* and others^34–36^. However, no information is available on *P. gossypiella,* in this regard. Our study showed that, thermal stress on F_1_ generation was evident and varied with different stages. The developmental duration of larval and pupal decreased with an increase in temperature; whereas, no significant differences were recorded in pupal weight and other reproductive parameters. These results suggest that, the impact of thermal stress was significant on the immature stages, but relatively less on the pupae and adults in F_1_ generation as compared to their parent (F_0_). It indicates that, progenies of *P. gossypiella* can overcome their parental thermal stress gradually, as it progresses to next developmental stage. Our data was consistent with other studies on other species like *Frankliniella occidentalis*, *Drosophila serrata*, and *Aphidius ervi,* which showed that, the impact of thermal stress on F_1_ generation was evident at younger stage and lower at the matured stage^37–39^. whereas, a study on *S. frugiperda* reported that, the persistent effect can be observed up to F_2_ generation^36^*.* Cell damage was evident when *Saccharomyces cerevisiae* was exposed to higher temperature, resulting reduction in ethanol production^41^. Therefore, insects can alleviate the thermal stress and steadily recover over generations by compromising some developmental traits^39^.

In conclusion, *Pectinophora gossypiella* emerges as a significant cotton pest with a notable ability to establish itself in diverse environments. Our study underscores the pivotal role of temperature in shaping the developmental and biological aspects of *P. gossypiella* across various temperature regimes. Optimal conditions for egg and larval development, as well as overall reproductive potential, were identified within the temperature range of 30 to 35 °C. Deviations from this range led to reduced egg hatchability and increased larval mortality. Additionally, the study highlighted the enduring impact of thermal stress on the F1 generation, with observable recovery as they progressed to subsequent stages. These insights hold promise for predicting field-level population dynamics and devising resilient management strategies to ensure sustainable cotton production in the face of climate challenges.

## Materials and Methods

### Pink bollworm culture

*Pectinophora gossypiella* infested bolls were collected from different cotton fields in the region of Raichur (16°12′ 2.9″ N and 77° 21′ 44″ E), Karnataka, India, in 2020. However, samples were collected with the appropriate permission from the authority and it comply with relevant institutional guidelines. The collected bolls were dissected to extract the third and fourth instar larvae of PBW. The larvae were collected into the rearing boxes (30 cm diameter × 45 cm height) containing fresh cotton bolls until pupation under laboratory conditions (27 ± 2 ^o^C, 55 ± 5% RH). The rearing boxes were checked daily and offered with fresh bolls as and when required. After pupation, males and females were sexed (based on gonad and anal pore)^40^ and transferred into plastic containers. On the day of adult eclosion, a pair of adults were transferred into mating chambers (30 × 45 cm) for oviposition and covered with muslin cloth. Moistened cotton with a 10% honey solution was provided as adult food, and fresh cotton twigs were offered to facilitate egg laying. Oviposited twigs were collected daily and replaced with new twigs. Further, the eggs were observed until hatching.

### Experimental design

After hatching, the neonates were transferred to rearing trays (Tarson, 45 × 30 cm, 96 wells made of polypropylene) containing an artificial diet and covered with a thin plastic lid. The diet was prepared as per the protocol^30,42^. Each tray consists of 30 larvae, and the trays were placed in six illuminated BOD chambers (Micro technologies) with constant temperatures of 20, 25, 30, 35, and 40 ± 1 ^o^C, with 65 ± 5% relative humidity and a photoperiod of 16:8 (16 h light: 8 h dark). The diet was changed and offered with fresh diet regularly till pupation. Pupae were sexed and placed in small plastic containers (10 × 15 cm) until adult emergence. Larvae from each tray were considered as replicates, and four replications were maintained in respective temperature conditions.

The life history traits were recorded from all individuals, which include the developmental time of egg, larvae and pupae. The survival rate of larvae, pupae, and pupal weight were counted at each temperature. After the emergence of adults, 30 pairs of moths were selected from each treatment and released a pair moths into plastic mating chambers (30 × 45 cm) separately, provided with cotton balls moistened in 10% honey as a food supplement and a fresh cotton twigs as ovipositional substrate. Cotton twigs were checked daily to record the egg count and replaced with fresh twigs whenever necessary. Those pairs did not lay eggs even 7 days after release was considered as unsuccessful mating. Further, the following observations were recorded, including length of pre-oviposition and oviposition periods, percent mating success, fecundity, and longevity of adults.

### Transgenerational effect

The carryover effect of temperature over F_1_ generation was investigated under laboratory conditions at 26 ± 2^o^C, 65% RH, and photoperiod of 16:8 h light: dark cycle. About 200 eggs from each treatment in the fecundity experiment (F_0_) were selected and the observations were recorded on developmental parameters including hatching percentage, developmental period of egg, larvae, and pupae. Each batch of larvae was reared independently on an artificial diet, as discussed above. After adult emergence, mating studies were conducted, as discussed above, with 30 pairs from each treatment. Further, reproductive parameters such as mating success (%), fecundity, and adult longevity were recorded individually.

### Statistical analysis

The observed data for all individuals at different temperatures were analysed and the life table was constructed by using ‘TWOSEX-MS Chart’ software^43,44^. According to the age-stage, two-sex life table, the following parameters viz., Age-stage-specific survival rates (*S*_*xj*_) = $\frac{{\text{n}}_{xj}}{{n}_{01}}$; Age-specific survival rate (*l*_*x*_) $=\sum_{j=1}^{m}{\mathbf{S}}_{\mathbf{x}\mathbf{j}}$; Age-stage-specific fecundity (*f*_*xj*_); Age-specific fecundity (*m*_*x*_) $=\frac{\sum_{j=1}^{m}{S}_{xj }{f}_{xj}}{{\sum }_{j=1}^{m}{S}_{xj}}$; Age-specific maternity (*l*_x_*m_*x*_); Age-stage-specific life expectancy (e_xj_) = $\sum_{j=1}^{m}\sum_{j=1}^{m}{S}_{ij}$; Age-stage-specific reproductive value (V_xj_) = $\frac{{e}^{-r(x+1)}}{{\text{S}}_{\text{xj}}} \sum_{i=x}^{n}{e}^{-r(i+1)}\sum_{j=y}^{m}{S}_{ij}{f}_{ij}$; Intrinsic rate of increase (r)- $\sum_{x=0}^{\infty }{e}^{-r(x+1)} {l}_{x} {m}_{x }=1$; Finite rate of increase (λ) = ${e}^{r}$; Net reproductive rate (R_0_) = $\sum_{x=0}^{\infty }{l}_{x} {m}_{x}$; Mean generation time (T)= $\frac{{\text{l}}_{n} {\text{R}}_{0}}{r}$ were studied. The means, standard errors and variances of the population parameters were estimated with 100,000 bootstrap replicates. Sigma plot 14.5 was used to create graphs. One-way ANOVA was used for analyse the thermal stress on F_0_ and F_1_ generation parameters such as developmental period of egg, larvae and pupae, pupal weight, pre-oviposition and oviposition periods, fecundity and longevity of adults using R software (Version 3.4.4)^45^.

## Data Availability

All the relevant data are presented in the manuscript. The datasets generated and analysed during the current study are available from the corresponding author upon request.

## References

[1] CABI. Invasive species compendium (2020) *Pectinophora gossypiella* (pink bollworm). https://www.cabi.org/isc/datasheet/39417#70AF7142-7A8B-4F36-A0BA-4F14FA270EED (2020).

[2] Naranjo, S. E., Butler, D. D. & Henneberry, T. J. *A bibliography of the pink bollworm, Pectinophora gossypiella (Saunders) (Bibliographies and Literature of Agriculture No. 136* (USDA Agricultural Research Service, 2002).

[3] Patil, S. B. Studies on management of cotton pink bollworm Pectionophora gossypiella (Saunders) (Lepidoptera: Gelechiidae). Ph.D. Thesis, University of Agricultural Sciences, Dharwad, Karnataka, (India) (2003).

[4] Naik, V. C., Jyothi, D., Dhabade, P. L. & Kranthi, S. Pink bollworm Pectinophora gossypiella (Saunders) infestation on Bt and non Bt hybrids in India in 2011–2012. *Cotton. Res. J.* **6**, 37–40 (2014).

[5] Vaissayre, M. Ecological attributes of major cotton pests: implications for management. In: Constable GA, Forrester NW (eds) Challenging the future. Proc. of the world cotton research conference I, Brisbane, Australia. CSIRO, pp. 499–510 (1995).

[6] Madhu, T. N. & Mohan, K. M. Effect of host plants on the oviposition preference of pink bollworm, Pectinophora gossypiella (Lepidoptera: Gelechiidae). *Ani. Biol.* **72**, 15–25 (2021). 10.1163/15707563-bja10064

[7] Chakravarthy, A. K., Naik, M. & Madhu, T. N. Arthropods on cotton: a comparison between Bt and non-Bt cotton. In *Economic and Ecological Significance of Arthropods in Diversified Ecosystems: Sustaining Regulatory Mechanisms* (ed. Chakravarthy, A. K.) (Springer, 2016).

[8] Rajashekhar, M. *et al.* Evaluation of integrated pest management module for pink bollworm, Pectinophora gossypiella (Saunders) and its economic analysis under farmer’s field conditions. *Int. J. Pest Manag.*10.1080/09670874.2022.2096269 (2022). 10.1080/09670874.2022.2096269

[9] Prabhulinga, T., Rameash, K., Madhu, T. N., Vivek, S. & Suke, R. Maximum entropy modelling for predicting the potential distribution of cotton whitefly Bemisia tabaci (Gennadius) in North India. *J. Entomol. Zoo. l Stud.* **5**, 1002–1006 (2017).

[10] Naik, V. C., Kumbhare, S., Kranthi, S., Satija, U. & Kranthi, K. R. Field-evolved resistance of pink bollworm, Pectinophora gossypiella (Saunders) (Lepidoptera: Gelechiidae), to transgenic Bacillus thuringiensis (Bt) cotton expressing crystal 1Ac (Cry1Ac) and Cry2Ab in India. *Pest Manag. Sci.* **74**(11), 2544–2554 (2018). 29697187 10.1002/ps.5038

[11] Naik, V. C. *et al.* Efficacy of novel insecticides and their combinations against pink bollworm, Pectinophora gossypiella (Saunders) (Lepidoptera: Gelechiidae) in cotton. *Int. J. Trop. Insect Sci.* **43**, 397–407 (2023). 10.1007/s42690-022-00939-8

[12] Intergovernmental Panel on Climate Change. Synthesis Report of Mitigation of Climate Change. Contribution of Working Group III to the Fifth Assessment Report of the Intergovernmental Panel on Climate Change. Cambridge University Press, Cambridge, United Kingdom and New York, NY, USA (2014).

[13] Atkinson, D. Temperature and organism size—A biological law for ectotherms?. *Adv. Ecol. Res.* **25**, 1–58 (1994). 10.1016/S0065-2504(08)60212-3

[14] Tobin, P. C., Nagarkatti, S. & Saunders, M. C. Phenology of Grape berry moth (Lepidoptera: Tortricidae) in cultivated grape at selected geographic locations. *Environ. Entomol.* **32**, 340–346 (2003). 10.1603/0046-225X-32.2.340

[15] Chen, C., Xia, Q. W., Xiao, H. J., Xiao, L. & Xue, F. S. A comparison of the life-history traits between diapause and direct development individuals in the cotton bollworm Helicoverpa armigera. *J. Insect Sci.* **14**, 19 (2014). 25373166 10.1093/jis/14.1.19 PMC4199537

[16] Sibly, R. M. & Atkinson, D. How rearing temperature affects optimal adult size in ectotherms. *Funct. Ecol.* **8**, 486–493 (1994). 10.2307/2390073

[17] Aguilon, D. J. D. & Velasco, L. R. I. Effects of larval rearing temperature and host plant condition on the development, survival, and coloration of African armyworm, Spodoptera exempta Walker (Lepidoptera: Noctuidae). *J. Environ. Sci. Manag.* **18**, 54–60 (2015).

[18] Nylin, S. & Gotthard, K. Plasticity in life-history traits. *Ann. Rev. Entomol.* **43**, 63–83 (1998). 9444750 10.1146/annurev.ento.43.1.63

[19] Jaworski, T. & Hilszczanski, J. The effect of temperature and humidity changes on insect development and their impact on forest ecosystems in the context of expected climate change. *For. Res. Pap.* **74**, 345–355 (2013).

[20] Rahmstorf, S. & Coumou, D. Increase of extreme events in a warming world. *Proc. Natl. Acad. Sci. U. S. A.* **108**(44), 17905–17909 (2011). 22025683 10.1073/pnas.1101766108 PMC3207670

[21] Hutchison, W. D., Butler, G. D. Jr. & Martin, J. M. Age-specific developmental times for pink bollworm (Lepidoptera: Gelechiidae): Three age classes of eggs, five larval instars, and pupae. *Ann. Entomol. Soc. Am.* **79**(3), 482–487 (1986). 10.1093/aesa/79.3.482

[22] Peddu, H., Fand, B. B., Sawai, H. R. & Lavhe, N. V. Estimation and validation of developmental thresholds and thermal requirements for cotton pink bollworm Pectinophora gossypiella. *Crop Prot.* **127**, 104984 (2020). 10.1016/j.cropro.2019.104984

[23] Garcia, A. G., Godoy, W. A. C., Thomas, J. M. G., Nagoshi, R. N. & Meagher, R. L. Delimiting strategic zones for the development of fall armyworm (Lepidoptera: Noctuidae) on corn in the state of Florida. *J. Econ. Entomol.* **111**, 120–126 (2017). 10.1093/jee/tox329 29267899

[24] Du Plessis, H., Schlemmer, M.L. & Van den Berg, J. The effect of temperature on the development of Spodoptera frugiperda (Lepidoptera: Noctuidae). *Insects*, **11**(4), 228 (2020). 10.3390/insects11040228 PMC7240686 32272548

[25] Mołoń, M. *et al.* Effects of temperature on lifespan of Drosophila melanogaster from different genetic backgrounds: Links between metabolic rate and longevity. *Insects* **11**(8), 470 (2020). 32722420 10.3390/insects11080470 PMC7469197

[26] Díaz-Álvarez, E. A. *et al.* Climate change can trigger fall armyworm outbreaks: A developmental response experiment with two Mexican maize landraces. *Int. J. Pest Manag.* **69**, 1–9 (2021).

[27] Barfield, C. S., Mitchell, E. R. & Poe, S. L. A temperature-dependent model for fall armyworm development. *Ann. Entomol. Soc. Am.* **71**, 70–74 (1978). 10.1093/aesa/71.1.70

[28] Henneberry, T. J. & Leal, M. P. Pink bollworm: effects of temperature, photoperiod and light intensity, moth age, and mating frequency on oviposition and egg viability. *J. Econ. Entomol.* **72**(4), 489–492 (1979). 10.1093/jee/72.4.489

[29] Ihsan, A. *et al.* Diet impacts on the biological aspects of pink bollworm, Pectinophora gossypiella (Lepidoptera: Gelechiidae) under controlled laboratory conditions. *PLoS One* **16**(11), e0258431 (2021). 34762679 10.1371/journal.pone.0258431 PMC8584967

[30] Madhu, T.N., Muralimohan, K., Arunkumara, C.G., Nagaraju, M.C. Effect of different nutrient diets on developmental and reproductive fitness of Pink bollworm, Pectinophora gossypiella (Lepidoptera: Gelechiidae) (2021).

[31] Zwaan, B. J., Bijlsma, R. & Hoekstra, R. F. On the Developmental Theory of Aging. 2. The Effect of Developmental Temperature on Longevity in Relation to Adult Body Size in Drosophila-Melanogaster. *Heredity* **68**, 123–130 (1992). 1548140 10.1038/hdy.1992.19

[32] Chen, Y. C., Chen, D. F., Yang, M. F. & Liu, J. F. The Effect of Temperatures and Hosts on the Life Cycle of Spodoptera frugiperda (Lepidoptera: Noctuidae). *Insects* **13**, 211 (2022). 35206784 10.3390/insects13020211 PMC8879478

[33] Madhu, T. N. *et al.* New Occurrence of the Spodoptera litura (Fabricius) (Lepidoptera: Noctuidae) Infestation on Cocoa in India. *J. Lepidopterists’ Soc.* **77**(2), 110–115 (2023). 10.18473/lepi.77i2.a4

[34] Mironidis, G. K. & Savopoulou-Soultani, M. Effects of heat shock on survival and reproduction of Helicoverpa armigera (Lepidoptera: Noctuidae) adults. *J. Therm. Biol.* **35**, 59–69 (2020). 10.1016/j.jtherbio.2009.11.001 28799914

[35] Sujatha, G. S. Studies on physiological responses of Spodoptera litura exposed to short and long-term thermal stress (Masters Dissertation, Division of Entomology ICAR-Indian Agricultural Research Institute, New Delhi) (2021).

[36] Reshma, R. *et al.* Transgenerational effects of thermal stress on reproductive physiology of fall armyworm Spodoptera frugiperda. *J. Pest Sci.* **96**(4), 1465–1481 (2023). 10.1007/s10340-023-01660-2

[37] Ismaeil, I. *et al.* Trans-generational efects of mild thermal stress on the life history traits of an aphid parasitoid. *PLoS One* **8**, e54306 (2013). 23405079 10.1371/journal.pone.0054306 PMC3566165

[38] Eggert, H., de Diddens-Buhr, M. F. & Kurtz, J. A temperature shock can lead to transgenerational immune priming in the Red Flour Beetle Tribolium castaneum. *Ecol. Evol.* **5**, 1318–1326 (2015). 25859336 10.1002/ece3.1443 PMC4377274

[39] Sun, L., Yabin, M. A., Honggang, L. I. & Zheng, C. The maternal efects of heat shock on biological parameters and ovaries ofFrankliniella occidentalis (Thysanoptera: Thripidae). *Eur. J. Entomol.* **116**, 212–220 (2019). 10.14411/eje.2019.023

[40] Ramya, R. S., Mohan, M. & Joshi, S. A simple method for sexing live larvae of pink bollworm, Pectinophora gossypiella (Lepidoptera: Gelechiidae). *Ani. Biol* **70**(1), 97–100 (2020). 10.1163/15707563-20191136

[41] Mueller, L. P., Santos, M. D. S. M., Cardoso, C. A. L. & Batistote, M. The effects of thermal and ethanolic stress in industrial strains of Saccharomyces cerevisiae. *Res. Soc. Dev.*10.33448/rsd-v9i10.9091 (2020). 10.33448/rsd-v9i10.9091

[42] Dharajothi, B., Naik, V., Kranthi, S., Kranthi, K. R. & Valarmathi,. Viable mass production method for cotton pink bollworm, Pectinophora gossypiella (Saunders). *J. Bas. Appl. Zool* **73**, 9–12 (2016). 10.1016/j.jobaz.2015.09.004

[43] Chi, H. Life-table analysis incorporating both sexes and variable development rates among individuals. *Environ. Entomol* **17**, 26–34 (1988). 10.1093/ee/17.1.26

[44] Chi, TWOSEX-MSChart: A Computer Program for the Age–Stage, Two-Sex Life Table Analysis. 2021, http://140.120.197.173/Ecology/prod02.htm (2021).

[45] R Core Team. R: A language and environment for statistical computing. R Foundation for Statistical Computing. https://www.R-project.org/ (2018).

