# Author Correction: Effect of temperature on the biological parameters of pink bollworm, *Pectinophora gossypiella* Saunders (Lepidoptera: Gelechiidae)

**DOI:** 10.1038/s41598-024-68546-w

**Published:** 2024-08-02

**Authors:** Madhu Tadagavadi Nagaraju, Kamanur Murali Mohan, Manikyanahalli Chandrashekara Keerthi, Tenguri Prabhulinga, Shivaji Thube, Vivek Shah, Hosam O. Elansary, Ihab Mohamed Mousa, Mohamed A. El-Sheikh

**Affiliations:** 1 ICAR-Central Institute for Cotton Research, Nagpur, Maharashtra 440010 India; 2 ICAR-Central Plantation Crops Research Institute, Regional Station, Vittal, Karnataka 574243 India; 3 University of Agricultural Sciences, Bangalore, Karnataka 560065 India; 4 ICAR-Indian Institute of Horticultural Research, Bengaluru, Karnataka 560089 India; 5 Department of Plant Production, College of Food and Agricultural Sciences, King Saud University, P.O Box 2460, 11451 Riyadh, Saudi Arabia; 6 Department of Botany and Microbiology, College of Food and Agricultural Sciences, King Saud University, P.O Box 2455, 11451 Riyadh, Saudi Arabia; 7 Department of Botany and Microbiology, College of Sciences, King Saud University, P.O Box 2455, 11451 Riyadh, Saudi Arabia

Correction to: *Scientific Reports* 10.1038/s41598-024-65241-8, published online 01 July 2024

In the original version of this Article Mohamed A. El-Sheikh was incorrectly affiliated with ‘Department of Botany and Microbiology, College of Food and Agricultural Sciences, King Saud University, P.O Box 2455, 11451, Riyadh, Saudi Arabia’.

The correct affiliation is listed below.

Department of Botany and Microbiology, College of Sciences, King Saud University, P.O Box 2455, 11451, Riyadh, Saudi Arabia

The original Article has been corrected.

